# Effect of HIV/AIDS and Malaria on the Context for Introduction of Zinc Treatment and Low-osmolarity ORS for Childhood Diarrhoea*

**Published:** 2008-03

**Authors:** Peter J. Winch, Kate E. Gilroy, Christa L. Fischer Walker

**Affiliations:** Department of International Health, Johns Hopkins Bloomberg School of Public Health, Room E5030, 615 North Wolfe Street, Baltimore, MD 21205-2103, USA

**Keywords:** Acquired immunodeficiency syndrome, Child health, Diarrhoea, HIV, Infant health, Malaria, Oral rehydration therapy, Osmolar concentration, Review literature, Zinc

## Abstract

Diarrhoea was estimated to account for 18% of the estimated 10.6 million deaths of children aged less than five years annually in 2003. Two—Africa and South-East Asia—of the six regions of the World Health Organization accounted for approximately 40% and 31% of these deaths respectively, or almost three-quarters of the global annual deaths of children aged less than five years attributable to diarrhoea. Much of the effort to roll out low-osmolarity oral rehydration solution (ORS) and supplementation of zinc for the management of diarrhoea accordingly is being devoted to sub-Saharan Africa and to South and South-East Asia. A number of significant differences exist in diarrhoea-treatment behaviours and challenges of the public-health systems between Africa and Asia. The differences in rates of ORS use are the most common indicator of treatment of diarrhoea and vary dramatically by and within region and may significantly influence the roll-out strategy for zinc and low-osmolarity ORS. The prevalence of HIV/AIDS and the endemi-city of malaria also differ greatly between regions; both the diseases consume the attention and financial commitment of public-health programmes in regions where rates are high. This paper examined how these differences could affect the context for the introduction of zinc and low-osmolarity ORS at various levels, including the process of policy dialogue with local decision-makers, questions to be addressed in formative research, implementation approaches, and strategies for behaviour-change communication and training of health workers.

## INTRODUCTION

Over a quarter-century has passed since the introduction of oral rehydration therapy (ORT) for diarrhoea in 1978 (1). ORT, including both prepackaged oral rehydration salts (ORS) and local recipes for home-fluids, remains the cornerstone of management of diarrhoea. ORT enables caregivers to manage dehydration in the home, decreases the need for intravenous fluids, and decreases rates of hospitalization and mortality. In 1980, diarrhoea was estimated to account annually for 4.6 million deaths of children aged less than five years (under-five children) (2), while recent estimates for 2003 attribute 18% or 1.9 million of 10.6 million annual deaths to diarrhoea, representing 3% of neonatal mortality and 17% of mortality in children aged 1–59 month(s) (3). Two—Africa and South-East Asia—of the six regions of the World Health Organization (WHO) account for approximately 40% and 31% deaths due to diarrhoea among children respectively, or almost three-quarters of the global annual deaths of children aged less than five years (under-five deaths) attributable to diarrhoea (3). This decrease in mortality is a great public-health success story, yet there has been limited or no decrease in rates of incidence of diarrhoea and morbidity (4). Improved case management is an important strategy to decrease the remaining 1.9 million childhood deaths attributable to diarrhoeal illness.

In May 2004, the WHO and United Nations Children's Fund (UNICEF) issued new recommendations for the management of all episodes of childhood diarrhoea, including new low-osmolarity oral rehydration salts (ORS) and supplementation of zinc for 10–14 days (5). When properly deployed, these two advances in treatment will decrease morbidity and enable further reductions in mortality due to diarrhoea. Low-osmolarity ORS continues to prevent and treat dehydration and also decreases stool volume by 25–30%, decreases the prevalence of vomiting by 30%, and decreases the need for unscheduled intravenous therapy by 30% (6-8). Supplementation of zinc for 10–14 days decreases the duration and severity of the diarrhoea episode and decreases morbidity from diarrhoea and pneumonia in the 2–3 months following treatment (9,10).

Zinc is an effective treatment for diarrhoea and resembles a modern pharmaceutical (tablets or syrup); therefore, zinc has the potential to reduce inappropriate use of antimicrobials for childhood diarrhoea through a replacement effect. Results of a community-based trial in Bangladesh showed that, in areas where zinc was introduced into the management of diarrhoea, inappropriate use of antibio-tics was significantly less than in control areas without zinc treatment (11). Preliminary evidence from Mali and India also suggests that the introduction of zinc treatment may reduce the unnecessary use of antibiotics for diarrhoea (12,13). Child-health and nutrition programmes will not only need to develop strategies to promote low-osmolarity ORS and supplementation of zinc, they can also work to discourage antibiotic use for simple diarrhoea through replacement with zinc.

There are now calls for a sustained effort to roll-out zinc and the new formulation of ORS and to ensure that it is in the hands of low-income and marginalized populations who most need it (14,15). Such efforts should also promote continued feeding, appropriate home-fluids, and breastfeeding.

Rates of ORS use for the management of diarrhoea vary greatly throughout sub-Saharan Africa and South Asia, reflecting regional variations in promotion and acceptance of ORS. High rates of incidence of HIV and malaria represent an added burden to already struggling health systems. This paper focuses on how these three factors—(a) current childhood diarrhoea-management practices, (b) prevalence of HIV, and (c) endemicity of malaria—affect the context of introduction of low-osmolarity ORS and supplementation of zinc at various levels: national policy, health programmes, health facilities, and households. We demonstrate that current rates of ORS use, HIV/AIDS, and malaria might affect the process of policy dialogue with local decision-makers, dictate questions to be addressed through formative research, and influence strategies for behaviour-change communication and training of health workers.

## SETTINGS FOR INTRODUCTION OF ZINC AND NEW ORS

For the purpose of this paper, countries in these two regions have been classified by rates of ORS use and rates of prevalence of HIV/AIDS and malaria (Table 1). These classifications are not intended to be comprehensive, but rather to illustrate the range of variation among countries and states in these two regions. For this reason, many, if not most, countries do not fit neatly into any one of the five settings described in Table 1.

**Table 1:** Public-health settings* for the roll-out of low-osmolarity ORS and zinc supplementation for management of diarrhoea in sub-Saharan Africa and South Asia

Characteristics	Setting 1	Setting 2	Setting 3	Setting 4	Setting 5
	Sahel Region Low ORS use, high burden of childhood malaria	Southern Africa High ORS use, high burden of HIV	Eastern and southern Africa Medium to high ORS use, high burden of HIV, and childhood malaria	South Asia Medium-high under-five mortality, low ORS use	South Asia High ORS use
Examples	Mali, Chad, Niger, Burkina Faso	South Africa, Namibia, Botswana	Uganda, Tanzania, Zambia, Malawi	Bihar State and Uttar Pradesh States, India	Bangladesh
<5 mortality rate/1,000 livebirths	175–200	60–80	110–180	100–125	60–80
Child health profile, Lancet series 2003	Profile 2	Profile 5	Profile 4	Profile 1	Profile 1
HIV prevalence	1–6%	20–30%	4–20%	<1%	<1%
Malaria transmission	Endemic in areas with adequate rainfall	Limited areas of transmission	Endemic in areas with adequate rainfall	Limited areas of transmission	Limited areas of transmission
Rates of ORS use	10–20%	50–70%	30–60%	10–20%	>60%
Availability of ORS sachets	Often only available in health facilities and pharmacies	Widely available through public and private sectors	Widely available through public sector, less in private sector	Widely available through public and private sectors	Widely available through public and private sectors
Production of ORS sachets	Often imported, some local production	Multiple producers, many flavours and brands	Some local production, sometimes imported	Multiple producers, many flavours and brands	Multiple producers, many flavours and brands
Marketing of ORS sachets	Limited private sector marketing, limited MoH promotion	Some private sector marketing, some MoH promotion	Some private sector marketing, some MoH promotion	Visible marketing of different brands, some MoH promotion	Visible marketing of different brands, some MoH promotion
Population density	Generally low	Low density in rural, large urban population	Low in rural areas, growing urban population	High	High

*Settings are not comprehensive; many countries do not fit one of these settings MoH=Ministry of Health; ORS=Oral rehydration salts

- Countries in this first category are primarily francophone, in the Sahel region of West Africa, and have among the highest levels of under-five mortality in the entire world (150–200/1,000 livebirths), low rates of ORS use (10–20%), high levels of malaria-related morbidity and morta-lity, relatively low but increasing prevalence of HIV, and relatively poor access to government health facilities. The 2003 Lancet child-survival series describes five profiles or patterns of under-five mortality in countries with high rates or contributing large numbers of under-five deaths (16). These countries were classified in Profile 2 in this typology of countries by under-five mortality pattern (16).
- This setting includes countries of southern Africa characterized by high ORS use, lower but still considerable under-five mortality (60–70/1,000 livebirths), overall high prevalence of HIV (over 20%), and highly seasonal and focal transmission of malaria, resulting in a relatively low burden of malaria-related disease. These countries were assigned to Profile 5 in the 2003 Lancet series (16).
- Countries in this setting include nations of eastern and southern Africa, falling in Profile 4 of the 2003 Lancet series (16), with medium-to-high under-five mortality (110–180/1,000 livebirths), medium-to-high ORS use (30–60%), overall medium-to-high prevalence of HIV (4–20%), and extensive malaria-related mortality and morbidity.
- This category includes countries of South Asia corresponding to Profile 1 in the Lancet series (16), with medium high under-five mortality (100–125/1,000 livebirths), low ORS use (10–20%), and a relatively small burden of disease attributable to HIV/AIDS and malaria compared to the countries mentioned above.
- The final setting is represented by parts of South Asia with lower but still considerable under-five mortality (60–80/1,000 livebirths), high ORS use (over 60%), and a relatively small burden of disease attributable to HIV/AIDS and malaria (14,17).

## CURRENT RATES OF ORS USE

ORS and ORT have been recommended for the treatment and prevention of dehydration since the late 1970s (1). Since the early promotional campaigns, some countries have placed more emphasis more on recommended home-fluids, while others have vigorously promoted and made available ORS sachets through the public or private sectors, or both (1); the definitions of appropriate home-fluids also vary greatly across countries. For these reasons, comparison of rates of ORS use between countries, states, or even regions does not adequately describe whether management of diarrhoea in the home is better in one place versus another. Nevertheless, since it is expected that low-osmolarity ORS and zinc will be distributed through the same mechanisms as the currently-available ORS sachets, rates of ORS use provide key information for the planning of strategies for the introduction of new diarrhoea-treatment guidelines.

Table 2 shows rates of ORS use based on the demographic and health surveys (DHS). There is a tremendous variation within region in both South and South-East Asia. Low levels of ORS use are observed throughout West Africa, except Ghana and Guinea. Reasons for low levels of ORS use in West Africa include greater promotion of home-available fluids than ORS in some countries, user-fees, and charges for products, such as ORS sachets in health facilities or community pharmacies operating under the principles of the Bamako Initiative (18), and poor geographic access to health facilities and pharmacies (19). High levels of ORS use were observed across southern Africa (Namibia, South Africa, and Zambia) and to some extent continuing up into east Africa (Tanzania and Uganda).

**Table 2:** Percentage of children with diarrhoea in the last two weeks who received ORS in sub-Saharan Africa and South and South-East Asia as measured by DHS surveys, 1998–2006

ORS-use rates (%)	Sub-Saharan Africa country and year of DHS survey (%: Country and year of survey)	South and South-East Asia country and year of DHS survey (%: Country and year of survey)
<20	12.4: Madagascar 2003/2004 13.1: Ethiopia 2000 13.6: Rwanda 2000 14.0: Mali 2006 15.1: Chad 2004 16.8: Cameroon 2004 17.1: Togo 1998 17.6: Niger 2006 18.2: Nigeria 2003 18.9: Burkina Faso 2003	17.8: Cambodia 2000
20–29	20.4: Senegal 1999 22.6: Mauritania 2000/2001 23.6: Côte d'Ivoire 1998/1999 24.8: Gabon 2000 29.2: Kenya 2003	28.8: India 1998–1999
30–39	33.5: Uganda 2000/2001 34.5: Guinea 1999 38.6: Ghana 2003	32.2: Nepal 2001 38.8: Pakistan 1990/1991 35.5: Indonesia 2002/2003
40–49	44.7: Eritrea 2002 47.9: Malawi 2000 48.5: Mozambique 2003	40.0: Viet Nam 2002 42.2: The Philippines 2003
50–59	51.2: South Africa 1998 53.2: Zambia 2001/2002 53.9: Tanzania 2004–2005	
60–69	61.1: Namibia 2000	67.7: Bangladesh 2004

DHS=Demographic and health survey; ORS=Oral rehydration salts

In South and South-East Asia, the lowest levels of ORS-use were observed in Cambodia and in the Hindi-speaking States of the Gangetic Plain (Uttar Pradesh and Bihar), while the highest levels were observed in Bangladesh, Viet Nam, The Philippines, and the smaller Indian States, such as Kerala, Manipur, and Goa. Factors contributing to lower levels of ORS-use in parts of South Asia include low levels of knowledge and awareness of ORS among some groups (20) and poor access to health services because of poverty and social marginalization of certain caste and ethnic groups (21).

Where rates of ORS use are high, ORS sachets are generally widely available, affordable, or free of charge (22) and their value for prevention and treatment of dehydration widely recognized (23,24). In these settings, formative research might examine current sources of ORS and consumer preferences for ORS and other treatments of diarrhoea and use this information to take advantage of existing distribution channels for the introduction of zinc supplementation. Where ORS is available free of charge, it is more likely that zinc may be available free of charge which will accelerate rates of uptake, especially among the very poor. In addition, locations with already high rates of ORS use may be in the best position to benefit from expanded private-sector social marketing campaigns for zinc and new ORS. In contrast, low rates of ORS use are associated with low awareness and knowledge of ORS among caregivers (25), poor availability at the community level (26), perceived high cost of ORS, or the misconception among both prescribers (27,28) and consumers (29) that antibiotics, antidiarrhoeals, or other treatments are effective medications for diarrhoea. These different factors, and how approaches to promote zinc and the new ORS can be adapted to these difficulties, should be examined through formative research.

The availability of and access to ORS sachets vary greatly between regions and can have a significant impact on use-rates. In many Asian countries, ORS sachets are available through various channels, such as health facilities, general stores, and medicine shops (22), and population densities are higher, all of which favours better geographic access to the product. The private Asian market has led to the widespread availability of ORS sachets and a competing private sector that does not depend on donor funds for promotion campaigns. The competition has led to a wide array of products boasting various flavours and brand names, although formulations are not always in accordance with the recommendations of WHO (30). Where ORS is largely produced within country, governments may be able to control how fast changes to the new formulation of ORS take place through policies requiring companies to produce the new formulation of ORS. It will be critical that governments take steps to ensure that low-osmolarity ORS sachets are supplied to both public and private sectors. Additional education to teach healthcare providers and consumers about the added value of the new formulation should accompany changes in policy and regulation.

Across southern Africa, ORS sachets tend to be readily available through both public and private channels, whereas in West Africa, ORS sachets are frequently available only at health facilities and pharmacies. These are few in number, making it difficult for people to access ORS, particularly in the rainy season. With the introduction of zinc and new ORS, strong advocacy should be carried out for policies which promote the increased access and availability of these products through private and community distribution channels. African countries also tend to not have as many local manufacturers of ORS as Asian countries do, therefore may only have one public-sector supplier and no private-sector ORS market. The adoption of the new formulation of ORS may be dependent upon the rate at which exporters of ORS adapt to the new formulation. In some cases, suppliers of ORS are producing both original and low-osmolarity ORS, but are only providing the new formulation when requested, thus leaving the choice of which formulation up to the procuring body.

## POTENTIAL EFFECTS OF MALARIA AND HIV/AIDS

The epidemiology of malaria and HIV/AIDS differs greatly across the five settings, with rates of childhood malaria highest in Setting 1 and 3 and HIV/AIDS taking the greatest toll in Setting 2 and 3. The presence of these diseases can affect the receptiveness of a given setting to introduction of zinc and low-osmolarity ORS and the feasibility of attaining an adequate coverage with these interventions (Table 3). The following paragraphs review the potential effects across five categories of considerations, as summarized in Table 3: (a) Policy, (b) Commodities and procurement, (c) Personnel, (d) Quality of care, and (e) Home-care.

**Table 3:** Effects of malaria and HIV/AIDS on introduction of zinc and low-osmolarity ORS for diarrhoea

Policy Effects on policy-makers and the process of policy dialogue	Competition for attention of policy-makers where HIV and malaria demand a large portion of their time Low priority assigned to diarrhoeal diseases compared to HIV and malaria Concern that zinc treatment might affect clinical course of AIDS
Commodities Supply of zinc and new ORS	Production, cost, and distribution of AIDS antiretroviral therapy, tools for HIV prevention and control, malaria combination treatment, and malaria-prevention tools may take priority over addressing supply of low-osmolarity ORS and treatment with zinc Competition with HIV and malaria drugs, tools, and vaccines for budget allotments Pharmaceutical companies may see greater potential for profit in production of antimalarial and antiretroviral treatments
Personnel Availability of human resources in the health sector	Competition for scarce human resources from other disease-control programmes Competition for time of health workers: training courses, meetings, supervision Morbidity and mortality in health workers due to AIDS or malaria
Quality of care Quality of case management by health workers	Clinical presentation of diarrhoea is more complex in children with HIV Confusion/overload in health workers relating to new guidelines for case management especially if they are not integrated with existing guidelines Emphasis by health workers often placed on management of fever/malaria than management of other illnesses
Home-care Home management of diarrhoea by parents or other caretakers of young children	Sickness or death of parents from AIDS or malaria results in households headed by elderly caregivers or children, difficult for these caregivers to absorb new guidelines for the management of diarrhoea Diarrhoea might be perceived as symptom of malaria (12) rather than a separate condition meriting specific treatment Results of formative research in southern Mali shows that health workers are likely to over-diagnose malaria giving antimalarials to a wider range of childhood illnesses, including diarrhoea Results of a pilot study in Mali showed that multiple treatments were beyond the financial means of families and difficult to administer correctly

ORS=Oral rehydration salts

### Policy

Prior to the introduction of zinc and low-osmolarity ORS for diarrhoea, policy-makers and decision-makers in the Ministry of Health will review the recommendations and evidence supporting their introduction and discuss options for implementation. High rates of HIV/AIDS and malaria may greatly complicate this process. Policy-makers are currently engaged in discussions about recommendations for the treatment of HIV and malaria, financing strategies for these expensive treatments, and how to mitigate the impact of HIV/AIDS on communities, including provision of care for orphans and other vulnerable children. This may leave decision-makers with little time to deliberate on new diarrhoea-management policies. Furthermore, in the presence of endemic malaria or high prevalence of HIV, diarrhoeal disease may be regarded as a relatively unimportant public-health problem. Finally, there may be concerns that treatment with zinc could adversely affect the clinical course of AIDS, although data have shown that supplementation of zinc is safe and effective when given for diarrhoea in HIV-1-infected children (31).

### Commodities and procurement

Adaptation of new diarrhoea-management guidelines will likely be most successful where low-osmolarity ORS and zinc will be widely available either free of charge or at a nominal cost. Past experience has demonstrated that where fees for treatments are instituted, coverage can be negatively impacted, especially among poorer families (32). In Setting 1, 2, and 3, issues relating to procurement or local production, cost and distribution of AIDS antiretroviral therapy, combination treatment of malaria, mosquito nets, insecticides, and other products may take priority over addressing issues of supply for low-osmolarity ORS and supplementation of zinc. Finally, pharmaceutical companies may see a greater potential for profit in production and marketing of antimalarials, antiretrovirals, antibiotics, or inappropriate antidiarrhoeals than in production of zinc. Zinc supplements do not require advanced technology, thus production at the local level is possible. However, companies may be hesitant to start product development prior to having a commitment from the government that there will be a market for the supplements. Because the new formulation of ORS requires only a change to the formulation, production of the low-osmolarity ORS requires only a decision on the part of the manufacturer to make the switch.

### Personnel

The availability of trained personnel can be impacted by high rates of HIV/AIDS and malaria, either directly by increasing rates of morbidity and mortality among qualified health workers, or indirectly through effects on health systems. Many interventions to reduce the burden of HIV/AIDS and malaria are being implemented through categorical (disease-specific) control programmes. Categorical programmes can weaken health systems by hiring already scarce personnel away from existing services into new categorical programmes, or offering specific courses, meetings, and other activities that take away from general patient care (33), although there is some evidence that categorical programmes may improve the management of health systems and community relations (34). In these areas, the introduction of zinc and new ORS should focus on strengthening existing primary-care and community-care infrastructure through training of personnel and reinforcing the integration of management of diarrhoea within the overall health system.

### Quality of care

Complex clinical presentations of diarrhoea among HIV-positive young children require greater skills to manage appropriately. Acute diarrhoea occurs more often in HIV-infected children, and episodes tend to be more severe and persistent (35,36) and are much more likely to lead to death (35). Furthermore, HIV-positive children are more likely to have concurrent symptoms of dehydration and fever (37) or co-morbidities, such as pneumonia (36), leading to a greater proportion of episodes requiring care at higher-level facilities, rather than in homes or in the community. Therefore, in regions with significant prevalence of childhood HIV, training modules for health workers, job aids, and reporting forms relating to management of diarrhoea must be adapted to account for more severe presentations of diarrhoeal illness among HIV-positive children.

Although there is little scientific evidence supporting biological interaction of malaria and diarrhoea (38-40), the coincidental occurrence of malaria and diarrhoea concurrently is well-documented, and children often present to healthcare providers with symptoms of both diarrhoea and fever or history of fever (40,42,43). In highly malaria-endemic countries, a typical recommendation is for all children aged less than five years presenting with fever or history of fever to be presumptively treated with an antimalarial (44). Therefore, in these areas, children presenting with fever and diarrhoea will need to receive zinc, ORS, and antimalarial treatment. Efforts will be needed to promote understanding of these new treatment guidelines in both formal and informal sectors.

### Home-care

*Household structure***:** Thus far, pilot interventions introducing supplementation of zinc and low-osmolarity ORS have relied upon community education on management of diarrhoea to increase treatment-seeking from public and private sources and, hence, improve management of diarrhoea. In countries where prevalence rates of HIV are high, severe illness and death among young adults have led to an enormous increase in the number of households headed by parents incapacitated by illness, elderly caregivers, or older children (45,46). Severe illness or absence of an adult caregiver can impact both home management and care-seeking behaviour for episodes of childhood diarrhoea. In Zaire, among HIV-negative children, those whose mothers were HIV-positive were two times more likely to develop persistent diarrhoea than children of uninfected mothers; HIV-negative children whose mothers died of HIV infection were 10 times more likely to develop persistent diarrhoea (35). Limited evidence from Uganda suggests that child-headed households have less knowledge about mild and severe malaria and are significantly less likely to seek care from health facilities and are more likely to use herbal remedies to treat malaria than adult-headed households (47). In such a setting, community-level infrastructure established to facilitate access to treatment of AIDS and mitigate the effects of AIDS may also be an effective platform for the promotion of improved management of diarrhoea and a way to reach vulnerable children.

*Perceptions of care-givers about childhood diarrhoea***:** Diarrhoea is often considered not as severe or life-threatening as malaria or HIV/AIDS, thus negatively affecting care-seeking and treatment patterns. Results of our work in Mali showed that parents commonly perceived that diarrhoea is a symptom caused by malaria (12). This misconception results in many children with diarrhoea and fever only receiving treatment of malaria with no recommendation for ORS or home-fluids (12). Therefore, during the introduction of zinc in these areas, communication campaigns should also focus on the potential severity of diarrhoea as an illness and cause of child death, not merely a symptom of other illnesses.

When any new treatment is introduced, there is the risk that it may displace treatments being used previously. This is both an opportunity and a challenge. In the case of inappropriate antibiotics and antidiarrhoeals, zinc may successfully displace these treatments. However, in countries where malaria is endemic, it is important to ensure that zinc is not perceived as a replacement for antimalarials. Children who present with both fever and diarrhoea should receive an antimalarial drug, zinc, and ORS/ORT. In a pilot introduction of zinc for the management of diarrhoea in Mali, it was observed that many children with both fever and diarrhoea received zinc and ORS but did not receive an antimalarial; families and community health workers viewed zinc as a medicine sufficient to treat both the illnesses (48). Remedial immediate actions were taken to improve and integrate community and home management of diarrhoea and malaria. New first-line artemisinin-based combination therapies are expensive, and their cost may prove prohibitive to families (49,50); families may perceive the new zinc treatment as a lower-cost alternative to artemisinin combination therapy for children with diarrhoea and fever. Training and communications materials for the introduction of zinc and new ORS must be integrated with childhood malaria-management strategies to ensure that both the diseases are adequately treated. Additionally, communication strategies to introduce new treatments for malaria and diarrhoea must work to create a distinct identity for zinc tablets and be coordinated in their choice of product names, logos, and explanations of properties of medicines. Monitoring the treatment patterns for both diarrhoea and malaria will be important early on to ensure that zinc is replacing unnecessary antibiotics, but not replacing antimalarials.

## CONCLUSIONS

In this paper, we have argued for the importance of considering rates of ORS use, endemicity of malaria, and rates of prevalence of HIV when planning for the introduction of zinc supplementation and low-osmolarity ORS for childhood diarrhoea. Research and evaluation of zinc supplementation thus far have focused on clinical outcomes with regard to HIV/AIDS and malaria, i.e. efficacy and safety measures. These are critical questions to address, but the vast impact that HIV/AIDS and malaria can have on health systems and how this will impact the process of revitalizing diarrhoea-control efforts also merit consideration and further study. In this paper, we have demonstrated that, when planning for the introduction of zinc and low-osmolarity ORS in a country where HIV/AIDS and/or malaria are important public-health problems, it is absolutely crucial to examine the direct and indirect effects the presence of these diseases may be having on (a) the policy environment and policy-makers, (b) the supply chain for medications and other health-related commodities, (c) the availability of human resources in the health sector, (d) the quality of case management by health workers, and (e) home management of diarrhoea by parents (Table 3).

Considering only rates of ORS use, endemicity of malaria, and rates of prevalence of HIV, the most favourable context for the accelerated introduction of zinc and low-osmolarity ORS will be where current rates of ORS use are high, and the burden of disease attributable to HIV/AIDS and malaria is low. This situation is represented by Setting 5 in Table 1. The overall environment for the introduction of zinc and low-osmolarity ORS is favourable due to the high awareness of the impact of diarrhoeal disease in the public-health community, and good local capacity for production and promotion of zinc and ORS.

Setting 1 and 4, where rates of ORS use are the lowest, may present the greatest challenges to introduction of these new treatments. If the lowest rates are due to ORS being unavailable, efforts may be needed to both promote local manufacturing of zinc and ORS and ensure that these products are available through multiple public and private distribution channels. Although the burden of disease attributable to HIV is currently lower compared to other countries, these regions have difficulties in achieving high coverage with other critical public-health interventions. Thus, achieving coverage of zinc and low-osmolarity ORS may require additional technical assistance to overcome challenges. The francophone countries in Setting 1 are characterized by high levels of under-five mortality, weak health services, poor transport, and limited ability of governments to implement policies and provide services (51). Under these conditions, the introduction of new diarrhoea-management guidelines may have the most success if coordinated with other child-health initiatives, such as UNICEF's Accelerated Child Survival and Development (ACSD) in West Africa and other large-scale programmes. In Setting 4, communication and transport infrastructures are far better than in Setting 1, but mortality levels remain high because it is difficult to ensure that health and nutrition interventions are delivered to the poorest wealth quintiles and marginalized groups. The Government of India and State governments are actively implementing programmes to overcome the constraints to decreasing under-five mortality, especially in challenging regions. Again, efforts need to be made to integrate the introduction of new diarrhoea-management guidelines with these initiatives.

In Setting 2 and 3, considerable progress has been made in strengthening health systems, and this is reflected in the high rates of ORS use and lower levels of under-five mortality compared to many other parts of Africa. HIV/AIDS threatens to reverse many of these gains and is a constant concern in the minds of policy-makers, health workers, and communities. In Setting 3, the challenge of HIV/AIDS is overlaid on the persistent high malaria-related burden of disease. In Setting 2 and 3, strong advocacy before policy-makers for investments to improve case management of diarrhoea is required. At the household and community levels, promotion of appropriate management of diarrhoea should be complemented by adequate care and support for orphans and other vulnerable children. As with Setting 1 and 4, we need to seek new ways of integrating ongoing efforts of new diarrhoea-management guidelines directed at HIV/AIDS and malaria through better training and supervision of health workers and other measures to improve quality of care.

It is widely appreciated that national strategies to introduce zinc for the management of diarrhoea and new low-osmolarity formulation of ORS must be adapted through formative research to take into account local understandings of diarrhoea and home-management practices. What is less well-appreciated is the enormous impact the other public-health problems, such as malaria and HIV/AIDS, can have on the environment for the introduction of these new treatments, as summarized in Table 3. Consideration of this situation necessarily widens the scope of formative and operations research and calls for us to carefully tailor strategies for advocacy of these new treatments and coordination of this introduction with other health initiatives.

## ACKNOWLEDGEMENTS

This paper was written with support from the Zinc Task Force with funding from the Bill and Melinda Gates Foundation and support from the Office of Health, Infectious Diseases and Nutrition, Global Health Bureau, United States Agency for International Development (Award No. HRN-A-00-96-90006-00).
